# Could Near Infrared Spectroscopy (NIRS) be the new weapon in our fight against Necrotising Enterocolitis?

**DOI:** 10.3389/fped.2022.1024566

**Published:** 2022-11-08

**Authors:** Claire Howarth, Jayanta Banerjee, Terence Leung, Narendra Aladangady

**Affiliations:** ^1^ Neonatal Unit, Homerton Healthcare NHS Foundation Trust, London, United Kingdom; ^2^ Neonatal Unit, Imperial College Healthcare NHS Trust and Imperial College London, London, United Kingdom; ^3^ Department of Medical Physics and Biomedical Engineering, Faculty of Engineering Sciences, University College London, London, United Kingdom; ^4^ Barts and The London School of Medicine and Dentistry, Queen Mary University of London (QMUL), London, United Kingdom

**Keywords:** NEC, biomarker, gut oxygenation, tissue injury, ischaemia, NIRS, regional oxygenation

## Abstract

There is no ideal single gut tissue or inflammatory biomarker available to help to try and identify Necrotising Enterocolitis (NEC) before its clinical onset. Neonatologists are all too familiar with the devastating consequences of NEC, and despite many advances in neonatal care the mortality and morbidity associated with NEC remains significant. In this article we review Near Infrared Spectroscopy (NIRS) as a method of measuring regional gut tissue oxygenation. We discuss its current and potential future applications, including considering its effectiveness as a possible new weapon in the early identification of NEC.

## What is known?

• Necrotising Enterocolitis (NEC) has significant morbidity and mortality
• There is currently no reliable and effective gut biomarker to predict NEC in routine clinical practice
• Near Infrared Spectroscopy (NIRS) is a non-invasive method of measuring regional tissue oxygenation
• Changes in splanchnic oxygenation could suggest the onset of gut tissue injury

## What is new?

• Near Infra-Red Spectroscopy (NIRS) could detect perfusion changes prior to development of NEC allowing opportunity for timely intervention, but further research is needed to examine if early intervention aided by gut NIRS measurements can improve outcomes
• Potentially a machine learning algorithm encompassing routine clinical, haematological, and biochemical parameters, in conjunction with gut related biomarkers and gut regional NIRS would be able to detect NEC prior to clinical manifestations

## Introduction

Necrotising Enterocolitis (NEC) occurs in 14% infants under 26 weeks gestation and 10% of babies born before 31 weeks gestation (1). Despite advances in neonatal care NEC continues to have a significant mortality and morbidity and with increasing survival of those more immature infants the population at risk of NEC is ever increasing. Although there has been a plethora of research studies examining the various blood and tissue biomarkers and their effectiveness in diagnosing gut tissue injury, none of the current biomarkers are in regular clinical use and most have some inherent difficulty with their measurement. Due to these difficulties, recent interest has turned to non-biochemical markers of gut hypoxia and ischaemia. This article reviews Near Infrared Spectroscopy (NIRS)—its current clinical use in Neonatology, its potential use in the identification and management of NEC, as well as exploring how effectively it could be implemented into routine clinical practice.

### Background and introduction to NIRS

Currently tissue perfusion and oxygenation of patients in Neonatal Intensive Care Units (NICU) is indirectly monitored using systemic blood pressure, heart rate, capillary refill time and evidence of end organ perfusion including lactate level, renal function, and urine output, which are often late signs of any disease process causing impaired perfusion. NIRS is a non-invasive method of contemporaneously measuring regional tissue oxygenation (rSO_2_) at the bedside. Changes in rSO_2_ measured by NIRS may reflect the balance between oxygen delivery and consumption (2).

### Principles of use

NIRS exploits the differential absorption of light of different biological compounds (e.g., haemoglobin) in the near-infrared region of the electromagnetic spectrum. The principle of how NIRS works in humans is best described by Cohn (3). NIRS monitors use near infrared light at wavelengths of relatively low optical absorption for oxyhemoglobin (HbO_2_) and deoxyhemoglobin (HHb), generally 700–850 nm, thus allowing near infrared light to penetrate and probe deeper into the tissue. The near infrared range is also where the absorption spectra of HbO_2_ and HHb are maximally separated with minimal overlap with that of water (980 nm) (4). NIRS can measure the ratio of HbO_2_ to HHb and the rSO_2_ can be derived using the equation: *rSO_2 _= HbO_2_/(HbO_2 _+ HHb)*.

Measurements using NIRS are specific to the region where the sensors are placed, and the rSO_2_ reflects perfusion and metabolism. They are not temperature or pulsatility dependent and because they are measured in “real-time” they could allow earlier detection of changes in oxygenation, blood flow and perfusion of the tissue. Tissue oxygenation measurements using NIRS are irrespective of systole or diastole; and as only 20% of blood volume is intra-arterial, measurements are primarily indicative of the venous oxygenation. The tissue microcirculation contains arteries, veins and capillaries meaning that the rSO_2_ represents a “weighted average” of SO_2_ in these compartments, with approximately 75%–85% of the signal originating from venules. As opposed to pulse-oximeters which subtract out non-pulsatile flow, NIRS monitors focus on the total light signal and the measured rSO_2_ reflects the balance of local tissue oxygen supply and demand meaning it is considered complementary to pulse oximetry (5).

Although regional tissue oxygenation measurements are venous weighted, by using peripheral pulse oximetry as a measure of arterial oxygenation, Fractional tissue oxygen extraction (FTOE) can be calculated. FTOE allows the estimation of the balance between oxygen delivery and consumption, at the site that the NIRS device is placed, can be calculated from the rSO_2_ and arterial oxygen saturation (SaO_2_) by the equation (6): *FTOE = [(SaO_2_−rSO_2_)/SaO_2_] × 100.* In addition, if cerebral and splanchnic oxygenation are measured simultaneously then the Cerebral Splanchnic Oxygenation Ratio (CSOR—which compares splanchnic oxygenation with cerebral oxygenation) can be calculated (6).

There are various NIRS monitors from different manufacturers which use different algorithms to measure tissue oxygenation. Different machines measure either the regional oxygen saturation (rSO_2_) or tissue oxygenation index (TOI), but both essentially reflect the ratio of HbO_2_ to total Hb (HbO_2_ + HHb).

### Current neonatal applications of NIRS

During the unique neonatal period following birth, the infant undergoes dramatic physiological changes during transition from intra- to extra-uterine life, involving significant changes in their haemodynamics which affect tissue oxygenation and would be reflected in rSO_2_. NIRS could be valuable in neonates; small size and thin layers covering tissues mean that NIRS measurements at depths of <5 cm can easily reach organs including the brain, kidney, and gut. This means that NIRS monitoring of these organs could yield invaluable physiological information not available in adults as these organs are comparatively less superficial (7). The additional advantage of NIRS is the ability of simultaneous oxygenation measurements of multiple sites (8, 9).

Studies involving cerebral oxygenation are outside the scope of this article but most research on the clinical application of NIRS focuses on cerebral measurements with many researchers having examined the effect of cerebral measurements on clinical outcomes (10–14). However, there is now increasing interest in its use for measuring splanchnic oxygenation. Although the number of studies available examining splanchnic oxygenation, especially over the last ten years, is large, they involve only a small number of infants (very large NIRS studies are still those involving less than 100 infants). The length of time the NIRS measurements were recorded also varies amongst studies and a lot of the studies only involve spot samples. Another issue with the current body of evidence is that the studies in the literature use different NIRS machines. The two most used machines are INVOS (rSO_2_) and the NIRO-300 (TOI); these measurements are different and therefore non-comparable between studies. Both these machines use different algorithms leading to intrinsically different oxygenation values. Furthermore, the sample volumes between the different machines also differ—the probe design is different meaning that the light source and detector spacing will be slightly different and, therefore, the tissue volumes that the probes sample will be different. Because the regional oxygenation is a weighted average of the arterial oxygenation and the venous oxygenation; if the sample volume is different the ratio of arterial to venous compartment volume could be slightly different, giving different results between the different NIRS machines. Consequently, when examining the literature surrounding NIRS it is important to identify which device was used and not to compare values between different monitors. To overcome this problem, it is more useful to compare percentage change from the baseline rather than the absolute value.

### Use of NIRS to identify splanchnic oxygenation and gut injury

In Neonatology NIRS more recently has been used to monitor splanchnic oxygenation (srSO_2_) to predict NEC and guide decisions regarding initiating feeds. Table 1 summarises the available neonatal studies on the use of NIRS to measure splanchnic oxygenation in neonates (9, 15–28). Table 2 summarises the literature available specifically for the use of NIRS for predicting or diagnosing NEC (15, 27, 29–39).

**Table 1:** Summary of the available literature on the use of Near Infrared Spectroscopy (NIRS) to measure splanchnic oxygenation in neonates.

Author and reference	Population studied	Study design (including measurement location)	Primary outcomes/findings
Normal ranges of splanchnic regional oxygenation			
Cortez et al. (15) (2011)	- *n* = 21 - preterm infants <30 w GA	- prospective observational cohort study - infants enrolled within 48 h of birth - continuous NIRS probe placed on left paraumbilical region for 14 days	- demonstrated feasibility of using NIRS continuously for 14 days - daily mean rSO_2_ values decreased over the first 9 days (*p* < 0.0001) followed by an increase from days 10–14 (*p* = 0.0061) - infants with feed intolerance had lower splanchnic regional saturations as compared with those tolerating feeds (*p* = 0.0043)
McNeill et al. (9) (2011)	- *n* = 14 enrolled but 2 excluded from analysis meaning *n* = 12 infants’ data used for analysis - healthy preterm infants (29–34w GA)	- continuous monitoring of abdominal (infra-umbilical), cerebral, and renal (right posterior-lateral flank) regional oxygenation using NIRS from the time of birth to 21 days of life - six infants were between 29 and 30 weeks gestation and 6 were between 32 and 33 weeks gestation. Some of the analysis was done in these subgroups	- abdominal regional oxygenation (32–66\%) was lower than cerebral (66%–83%) and renal (64%–87%) - cerebral and renal oxygenation decreased significantly over the first weeks of life (*p* < 0.01) - abdominal oxygenation decreased over week 1 of life and then increased up to day 21. The median nadir was at day 7 (range 3–9) for those born at 29–30 weeks and day 4.5 (range 3–8) for those born at 32–33 weeks - regional oxygenation variability was lowest for cerebral measurements and highest at the abdomen - abdominal variability decreased significantly over time (*p* ≤ 0.05)
van der Heide et al. (16) (2021)	- *n* = 220 - GA <32w and/or BW <1,200 g - excluded those with NEC/sepsis/died	- prospective study - over the first week after birth measured a daily 2 h mean of r_s_SO_2_ (infra-umbilical) to assess its associations with sex, GA, postnatal age (PNA), small-for-gestational age (SGA) status, patent ductus arteriosus, haemoglobin, nutrition, and head circumference at birth - used these factors to create a prediction model r_s_SO_2_ = 3.2 − 7.0 × PNA + 0.8 × PNA^2^ − 4.0 × SGA + 1.8 × GA.	- on day 1, the mean ± SD r_s_SO_2_ value was 48.2% ± 16.6. The nadir of r_s_SO_2_ was on day 4 (38.7% ± 16.6 smoothed line) to 5 (37.4%±17.3, actual data), after which r_s_SO_2_ increased to 44.2% ± 16.6 on day 7. - r_s_SO_2_ is lower in infants with a lower gestational age and in small-for-gestational age infants - gestational age, postnatal age, and small-for-gestational age status affect regional splanchnic oxygen saturation and need to be considered when interpreting regional splanchnic oxygen saturations using NIRS - authors provided a model so that reference values for infant regional splanchnic oxygen saturation can be computed with a formula: (r_s_SO_2_ = 3.2 − 7.0 × PNA + 0.8 × PNA^2^ − 4.0 × SGA + 1.8 × GA)
Splanchnic NIRS and sepsis			
Calderon et al. (17) 2016)	- *n* = 69 - animal study involving rabbits - 14 died, 15 control and 40 in the study group	- used NIRS to measure regional hepatic and splanchnic (infra-umbilical) oxygenation - study group injected with Escherichia coli toxin to see the effect of sepsis on abdominal perfusion and whether NIRS was a valid tool to measure this	- hepatic rSO_2_ had a significant reduction 60 min after the administration of the toxin (*p* = 0.034) although not significant when the overall reduction analysed - splanchnic rSO_2_ decreased earlier from 30 min after the administration of the toxin (*p* < 0.001)
Splanchnic NIRS and PDA Note other studies in this table comment on impact of PDA on NIRS readings, but was not their primary outcome (therefore this has been highlighted under each study where relevant)			
Meier et al. (18) (2006)	- *n* = 1	- case report - infant with large PDA whose mesenteric (midline abdomen below umbilicus) and cerebral rSO_2_ were measured pre and post PDA ligation	- pre-PDA ligation the infant had lower cerebral and mesenteric rSO_2_ which significantly increased post PDA ligation (*p* < 0.0001)
Petrova et al. (19) (2011)	- *n* = 38 - preterm infants <32w GA with confirmed PDA on echocardiogram	- prospective cohort study - NIRS used to measure cerebral (rSO_2_-C), renal (thoracolumbar) (rSO_2_-R) and mesenteric (midline region below umbilicus) (rSO_2_ -M) oxygenation for 60 min before pharmacological treatment of PDA	- no significant difference in rSO_2_-C or rSO_2_-R irrespective of respiratory support - in Infants with a large PDA on nCPAP, the rSO_2_-M was lower and mesenteric FTOE higher than those mechanically ventilated and those with moderate PDA - significantly higher proportion of infants with a moderate PDA were mechanically ventilated compared with large PDA group
Splanchnic NIRS and feeding Note other studies in this table comment on effect of feeding on NIRS readings, but this was not their primary outcome (therefore this has been highlighted under each study where relevant)			
Braski et al. (20) (2018)	- *n* = 52 - preterm infants with GA ≤32w and ≤12w of age	- prospective observational two-centre study involving preterm infants who were stable and tolerating enteral feeds - infants had cerebral and splanchnic (infra umbilical) rSO_2_ measured using NIRS continuously for 24 h - SCOR was subsequently calculated - mean regional oxygenation was calculated for 30 min prior to each feed, for the duration of each feed and 30 min after each feed	- average mean baseline SCOR decreased significantly during feeds (*p* = 0.043) - no significant difference in cerebral or splanchnic oxygenation during feeds although there was a trend to decreased splanchnic oxygenation during feeds - infants with lowest SCOR pre feeds had the largest decrease in SCOR with feeds
Corvaglia et al. (21) (2014)	- *n* = 30 - preterm infants <32w GA receiving NGT feeds at more than 100 ml/kg/day	- prospective observational study - -cerebral and splanchnic (infra umbilical) oxygenation measured using NIRS for 6 h while the infants had two feeds—one was bolus, and one was continuous feed	- no change in cerebral oxygenation over time regardless of feeding method - significant increase in splanchnic oxygenation after bolus feeds and a reduction in splanchnic oxygenation during continuous feeding (*p* < 0.0001)
Gillam-Krakauer et al. (22)(2013)	- *n* = 25 preterm infants less than 14 days of life enrolled but 7 withdrawn so 18 for analysis - GA <31 w - BW <1,500 g	- prospective observational study - -measured splanchnic regional oxygenation (infra umbilical using NIRS continuously for 3 days and measured SMA artery velocities using doppler USS - -compared change in SMA velocity from immediately before to 10 min and 60–120 min after feeding with change in the abdominal regional oxygenation - Spearman's rank correlation used to see if a significant association existed	- changes in splanchnic regional oxygenation was significantly associated with changes in systolic, diastolic, and mean SMA velocity from fasting to 60–120 min after feeding (*p* = 0.016, 0.021, 0.010) and from 10 min after a feed to 60–120 min after feeding (*p* = 0.009, 0.035, 0.032)
Martini et al. (23) (2018)	- *n* = 20 - preterm infants <34w GA with absent or reversed umbilical EDF Antenatally	- observational pilot study - continuous monitoring of splanchnic oxygen saturation (infra umbilical) (SrSO_2_) and cerebral oxygen saturation (CrSO_2_) at enteral feeding introduction until fully enterally fed (≥150 ml/kg/day) - monitoring took place 30 min before feeding until 3 h after feed	- -infants who developed gastrointestinal complications later demonstrated significantly lower SrSO_2_ (*p* = 0.02)
Studies involving splanchnic NIRS to measure the effect of RBCT/anaemia			
Bailey et al. (24) (2010)	- -*n* = 35 infants - preterm infants <37w - at least 5 days old at time of RBCT	- prospective, observational study - simultaneous measurement of cerebral rSO_2_ and splanchnic (in the midline below the umbilicus and above the pubic symphysis) rSO_2_ during RBCT (15 ml/kg RBCT given)	- cerebral rSO_2_ increased after RBCT and remained elevated for 12 h - splanchnic rSO_2_ also increased following RBCT although this increased after the rise in cerebral rSO_2_ - no correlation between Hb levels and cerebral rSO_2_ (r = −0.17, *p* = 0.36) or splanchnic rSO_2_ (r = −0.07, *p* = 0.72) before or following RBCT
Bailey et al. (28) (2012)	- *n* = 55 - preterm infants in first 5 days of life with Hb ≤11 g/dl	- prospective observational pilot study - measured cerebral and splanchnic oxygenation (just below the umbilicus) and then calculated the CSOR - CSOR was used as a marker for need for RBCT in preterm infants - groups divided into whether they were symptomatic or not from anaemia and whether improved or not after RBCT if it was given	- symptomatic patients who improved following RBCT had a low preceding CSOR (≤0.73) which improved following RBCT (*p* = 0.03) - symptomatic infants who did not improve following RBCT had higher CSOR values prior to RBCT
Banerjee et al. (64) (2016)	- *n* = 59 - preterm infants (≤34 w GA)	- prospective observational study - preterm infants receiving RBCT for clinical indication - infants divided into three groups based on postnatal age - a single operator measured the SMA peak systolic and diastolic velocities 30–60 min before and after RBCT - used NIRS with the probes placed in the hypogastrium in the midline above the symphysis pubis to assess splanchnic oxygenation (sTOI) 15–20 min, during and 15–20 min after RBCT	- after RBCT sTOI increased (*p* < 0.01) and FTOE decreased (*p* = 0.02) in all groups - sTOI correlated with SMA blood flow
Dani et al. (25) (2010)	- -*n* = 15 - preterm infants <30 w GA	- prospective observational study - NIRS was used to measure cerebral, splanchnic (infra umbilical), and renal (right of midline on the T10-L2 posterior flank) rSO_2_ during RBCT	- cerebral, splanchnic, and renal rSO_2_ increased following RBCT - associated decrease in FTOE in symptomatic anaemic preterm infants
Mintzer et al. (26) (2014)	- *n* = 10 - infants with BW <1,250 g	- observational pilot study - infants enrolled within 72 h after birth and monitored for 7 days - measured cerebral, renal (right flank), and splanchnic (infra umbilical) rSO_2_ in infants receiving “booster” RBCT - infants with similar VLBW with normal haematocrit and <10 ml/kg blood taken for routine blood samples were used as control infants for comparison	- rSO_2_ increased and FTOE decreased for each patient following “booster” RBCT (*p* < 0.05) - in all groups splanchnic rSO_2_ values were lower than cerebral and renal rSO_2_ - in all groups cerebral rSO_2_ was the highest (*p* < 0.05)
Sood et al. (27) (2014)	- *n* = 57 - preterm infants - median GA 27 w	- monitored cerebral and splanchnic (infraumbilical) rSO_2_ in preterm infants receiving RBCTs - defined 3 time points (pre RBCT—12 h prior, during RBCT and post RBCT—24 h after RBCT) and 3 groups (1 = no NEC within 7 days of RBCT, 2 = NEC within 7 days prior and 3 = NEC within 7 days after RBCT) - the 57 infants received 147 RBCTs (Group 1 = 120, Group 2 = 19, and Group 3 = 8)	- in group 1 and 2 rSO_2_ increased over RBCT periods - in group 3 rSO_2_ decreased over RBCT periods - RBCT, followed by a diagnosis of NEC, were characterised by lower heart rates pre-, during and post-RBCT, decline in sRSO_2_ and increase in cFTOE post-RBCT compared to RBCTs not associated with diagnosis of NEC - infants received RBCT who then developed NEC were characterised by higher variability in sRSO_2,_ post RBCT reduction in sRSO_2_ and lower CSOR values post RBCT compared to pre RBCT

BW, birthweight; CGA, corrected gestational age; crSO_2_, cerebral oxygenation; CSOR/SCOR, cerebral splanchnic oxygenation ratio; cTOI, cerebral tissue oxygenation index; ELBW, extremely low birthweight; FTOE, Fractional tissue oxygen extraction; GA, gestational age; Hb, Haemoglobin; HbF, Haemoglobin F; NEC, Necrotising Enterocolitis; NIRS, Near Infrared Spectroscopy; PDA, patent ductus arteriosus; RBCT, red blood cell transfusion; rSO_2_, regional oxygenation; SMA, superior mesenteric artery; sTOI, splanchnic tissue oxygenation index; TANEC, transfusion associated NEC; TR-NEC, Transfusion related NEC; VLBW, very low birth weight.

**Table 2:** Summary of the available literature on the use of Near Infrared Spectroscopy (NIRS) in predicting/diagnosing NEC in neonates.

Author and reference	Population studied	Study design	Primary outcomes/findings
Cortez et al. (15) (2011)	- *n* = 21 - preterm infants less than 30 weeks gestation enrolled (2 excluded from results)	- abdominal NIRS (left paraumbilical region) placed from within 48 h of birth for the first 14 days	- daily mean sRSO_2_ values decreased over first 9 days (*p* < 0.0001) followed by increase from day 10 to 14 (*p* = 0.0061) - sRSO_2_ was lower and FTOE higher in infants with feeding intolerance compared to those without (*p* = 0.0043) - higher sRSO_2_ and variability was associated with a healthy gut (*n* = 17) - neonates with NEC had low splanchnic rSO_2_s and decreased variability (*n* = 2) - very small study size—only two babies within their study cohort developed NEC
Fortune et al. (29) (2001)	- *n* = 40 - newborn infants - 10 with acute abdomens (4 NEC) - 29 controls - 1 hypoxic ischaemic injury	- prospective, observational cohort study - cerebral and splanchnic (infraumbilical) regional TOI measured using NIRS - calculated CSOR - measured prior to surgery/admission and then daily until discharge	- neonates with abdominal pathology had lower CSOR (*p* < 0.001) - CSOR detected the presence of intra-abdominal pathology with a sensitivity of 90% (56–100) and specificity of 96% (82–100) - if CSOR <0.75 intestinal ischaemia was identified with a PPV of 0.75 (0.43–0.95) and excluded with a NPV of 0.96 (0.81–1.0)
Gay et al. (30) (2011)	- *n* = 29 premature piglets - 3 developed NEC - 11 died prematurely - 15 served as controls	- serial abdominal (1 cm lateral to the umbilicus) NIRS recordings were taken of premature piglets who had received parenteral nutrition followed by enteral feeding - piglets monitored for developing NEC	- abdominal NIRS within 12 h of birth was significantly lower (*p* = 0.02) in infants who subsequently developed NEC compared with controls - for all time points measured, abdominal NIRS were significantly lower in the NEC group compared with controls (21% vs. 55%, *p* = 0.01). - -the authors drew a sensible conclusion that these lower regional oxygenation readings with abdominal NIRS in piglets with NEC represented intestinal ischemia-reperfusion injury—a well-known theory for the pathogenesis of NEC - also demonstrated that in healthy piglets, when oxygen levels decreased during apnoeas, there was a decrease in the abdominal NIRS oxygenation (r = 0.96) which increased again once the apnoea resolved, demonstrating a clinical correlation with the gut NIRS readings
Howarth et al. (31) (2020)	- *n* = 48 - preterm infants <30w gestation - median BW 884 (range 460–1,600) grams, median GA 26 + 3 (23 + 0–29 + 6) weeks	- Cerebral oximetry measurements were performed using a NIRS monitor weekly for 60 min allowing measurement of cTOI from first week of life to 36 weeks post conceptional age	- 276 NIRS measurements were completed, and 7 infants developed NEC - infants who developed NEC had significantly lower cTOI than those that did not (*p* = 0.011), even when adjusted for confounders including GA, BW, PDA, enteral feeds, gender, ethnicity, and Haemoglobin
Kalteren et al. (32) (2022)	- <32 w gestational age - *n* = 29 infants who received 58 RBCT - median GA 27.3 w	- prospective observational cohort study from March 2019 until December 2020 - measured urinary biomarkers for oxidative stress (8-isoprostane) and intestinal cell injury (I-FABP) shortly before and after RBCT - rsSO2 and rsSO2 variability were assessed simultaneously using INVOS 510°c oximeter placed in the infra umbilical region	- 6 out of 29 developed NEC after RBCT - Urinary 8-isoprostane and I-FABP increased nearly 2 fold following RBCT (median 282–606 pg/ml and 4,732–6,968 pg/ml, *p* < 0.01) - this increase was more pronounced in infants who developed NEC - Changes in I-FABP correlated with changes in 8-isoprostane (rho = 0.623, *p* < 0.01) - Lower rsSO2 variability, but not higher mean rsSO2 was associated with higher 8-isoprostane and I-FABP levels after RBCT - RBCT are associated with signs of associated with concomitant signs of oxidative stress and intestinal injury, parallel with lower variability in splanchnic oxygenation - authors postulated that this may represent the early pathogenetic process of transfusion-associated NEC
Le Bouhellec et al. (33) (2021)	- *n* = 45 - mean GA of 31 weeks - mean BW 1,486 g	- assessed the ability of NIRS to distinguish those neonates with NEC soon after symptom onset - prospectively collected NIRS measurements of abdominal (infra-umbilically on the central abdominal wall) and cerebral regional tissue oxygen saturation (r-SO_2_), with values masked by an opaque cover. - Two physicians, blinded to the NIRS data, determined whether the gastrointestinal symptoms were related to NEC 10 days after symptom onset.	- Gastrointestinal symptoms were related to NEC in 23 patients and associated with other causes in 22 - Analysis of the 48 h of monitoring revealed comparable abdominal r-SO_2_ and splanchnic-cerebral oxygenation ratio (SCOR) in patients with and without NEC (r-SO2: 47.3 [20.4] vs. 50.4 [17.8], *p* = 0.59, SCOR: 0.64 [0.26] vs. 0.69 [0.24], *p* = 0.51). - Results were unchanged after NIRS analysis in 6-hour periods, and restriction of the analysis to severe NEC (i.e., grade 2 and 3, 57% of the NEC cases). - in this small study, NIRS monitoring was unable to individualize NEC in premature infants with acute gastrointestinal symptoms.
Marin et al. (34) (2013)	- *n* = 8 - preterm infants receiving RBCT - TR-NEC infants were 24-29w GA and BW 705-1,080 g - non-NEC infants were 27.6-30w GA and BW 980-1210g	- infants divided into those with NEC post transfusion (TR-NEC, *n* = 4) and those without (non-NEC, *n* = 4) - measured cerebral and mesenteric lower abdomen) oxygenation patterns before, during and 48 h after RBCT using NIRS - alculated mean baseline rSO_2_ change and CSOR	- TR-NEC group received larger mean volumes of total blood than non-NEC infants - TR-NEC group showed wider fluctuation above and below baseline in oxygen saturations than the non-NEC group
Patel et al. (35) (2014)	- *n* = 100 - preterm infants <32w GA and BW <1,500 g enrolled - 8 with incomplete data excluded - divided into groups: infants with NEC (*n* = 14) and normal preterm infants without NEC (*n* = 78)	- 2 year prospective cohort study - abdominal (right lower abdomen) NIRS measurements - taken 5 min every day for the first week and then the same day once weekly for the next 4 weeks - compared between those with and without NEC	- mean abdominal rSO_2_ in healthy preterm infants during the first week of life was significantly higher than those who later developed NEC (77.3% ± 14.4% vs. 70.7% ± 19.1%, *p* = 0.002) - infants who developed NEC had a greater variation in abdominal rSO_2_ during feeding for first 2 weeks of life - authors suggested that a rSO_2_ of ≤56% increases the likelihood of later developing NEC (86% sensitivity, 64% specificity, 96% NPV and 30% PPV) - abdominal rSO_2_ of ≤56% was independently associated with a significantly increased risk of NEC (OR 14.1; *p* = 0.01) - infants with PDA had significantly lower rSO_2_ than those without (*p* = 0.023)
Schat et al. (36) (2016)	- *n* = 33 - preterm infants - median GA 28w - median BW 1,235 g	- prospective observational cohort study - 13 infants no NEC - 20 NEC (10 uncomplicated, 10 complicated—Bells stage 3B or death) - mean 8 hly cerebral, liver (right costal arch)and infraumbilical regional oxygenation in those infants with no NEC and those with complicated and uncomplicated NEC in the first 48 h after symptoms developed	- no difference between those with NEC and no NEC regional oxygenation levels in the first 24 h after symptom onset - no significant difference in the first 24 h after symptom onset in regional oxygenation between infants with no NEC and definite NEC - significantly lower cerebral, liver and infraumbilical levels in those with complicated NEC in the first 24 h after onset of symptoms compared with those infants with uncomplicated NEC - cerebral regional oxygenation ≤71% in first 8 h after symptom onset predicted complicated NEC with sensitivity 100% and specificity 80% - liver oxygenation ≤59% in first 8 h after symptom onset predicted complicated NEC with sensitivity and specificity of both 100%
Schat et al. (37) (2019)	- *n* = 30 - preterm infants <32 w GA - median GA 27.1 w - median BW 903 g	- case control study - 10 infants with NEC and 20 control infants matched for GA/BW/presence of PDA - cerebral and intestinal (infraumbilical) regional oxygenation measured using NIRS 2 h daily for first 5 days and then weekly until 5 weeks of life or until NEC developed	- cerebral oxygenation was significantly decreased in those that later went on to develop NEC - cerebral regional oxygenation <70% within the first 48 h of life developed NEC significantly more often than those with cerebral regional oxygenation ≥70% [OR 9 (95% CI 1.33-61.14)] - no difference in intestinal regional oxygenation measurements in those with NEC and without NEC in the first week of life
Sood et al. (27) (2014)	- *n* = 57 infants - median gestational age of 27 weeks - received 147 RBCTs.	- monitored cerebral and sRSO_2_ (infraumbilical) in preterm infants receiving RBCTs - defined three time points (pre RBCT—12 h prior), during RBCT and post RBCT—24 h after RBCT) - also defined 3 groups (1 = no NEC within 7 days of RBCT [*n* = 120], 2 = NEC within 7 days prior [*n* = 19] and 3 = NEC within 7 days after RBCT [*n* = 8]).	- in group 1 and 2 rSO_2_ increased over RBCT periods but in group 3 rSO_2_ decreased over RBCT periods - RBCT, followed by a diagnosis of NEC, were characterised by lower heart rates pre-, during and post-RBCT, decline in sRSO_2_ and increase in cFTOE post-RBCT compared to RBCTs not associated with diagnosis of NEC. - Infants received a RBCT who then developed NEC were characterised by a higher variability in sRSO_2,_ post RBCT reduction in sRSO_2_ and lower CSOR values post RBCT compared to pre RBCT - authors postulated that sRSO_2_ response to RBCT may potentially be a biomarker to identify infants more likely to develop TR-NEC after a RBCT
Stapleton et al. (38) (2007)	- *n* = 1	- case report - one infant with background of congenital heart disease who developed NEC - abdominal (midline below the umbilicus and above the pubic symphysis) and cerebral NIRS measurements performed 48 h after diagnosis of NEC was made	- initial abdominal NIRS readings showed low mesenteric rSO_2_ when compared with cerebral rSO_2_ (*p* < 0.0001) - after conservative medical treatment for NEC (NBM and IV antibiotics) mesenteric rSO_2_ improved compared with initial value
Zabaneh et al. (39) (2011)	- *n* = 2	- case report - 12-day-old growth restricted infant with NEC whose twin did not develop NEC - abdominal NIRS infra umbilical) measured 48 h after NEC diagnosis made and measured at irregular intervals - measurements compared with asymptomatic twin	- mesenteric rSO_2_ were reduced in the twin with NEC. - mesenteric rSO_2_ returned to similar level as asymptomatic twin after bowel resection.

BW, birthweight); CGA, corrected gestational age; ELBW, extremely low birthweight; EPO, Erythropoietin; GA, gestational age; Hb, Haemoglobin; I-FABP, Intestinal fatty acid binding protein; IFN gamma, interferon gamma; IL-1 β. interleukin 1 beta; IL-6, interleukin 6; IL-8 interleukin 8; IL-10, interleukin 10; IL-17, interleukin 17; L-FABP, liver fatty acid binding protein; NEC, Necrotising Enterocolitis; NIRS, Near Infrared Spectroscopy; PCA, post conceptual age; RBCT, red blood cell transfusion; TANEC, transfusion associated NEC; TNF-α, Tumour necrosis factor alpha;TR-NEC, Transfusion related NEC; VLBW, very low birth weight.

It is important to highlight that to date this has only been on limited numbers and there is an inherent problem with measuring srSO_2_ non-invasively using NIRS, in that the amount of gas (abdominal distension) and faecal content effects the measurement (27). In preterm infants these factors are constantly changing; respiratory support commonly given to preterm infants such as continuous positive airway pressure (CPAP) frequently causes abdominal distension, and in the first few days of life, they are known to not open their bowels regularly when they are on limited enteral feeds.

The validity of NIRS-derived srSO_2_ measurements for examining splanchnic perfusion and oxygenation has already been confirmed as srSO_2_ strongly correlates with gastric pH, serum lactate and systemic mixed venous saturations (2). In a clinical validation study of 59 preterm infants receiving blood transfusion compared with 12 control infants it was demonstrated that splanchnic NIRS is a reliable technique to inform changes in srSO_2_ following blood transfusion (40). A recent small study by Gillam-Krakauer et al. using Doppler confirmed that splanchnic NIRS reflects blood flow to the small intestine (22). In their study srSO_2_ was continuously measured for 3 days in stable infants born at 25–31 weeks gestation (*n* = 18) and they then compared changes in Superior Mesenteric Artery (SMA) velocity from immediately before to 10 min and 60–120 min after feeding with change in abdominal rSO_2_ over the same time. They reported that a change in abdominal rSO_2_ was significantly associated with change in systolic, diastolic and mean SMA velocity from fasting to 60–120 min after feeding (*p* = 0.016, 0.021, 0.010) and from 10 min after a feed to 60–120 min after feeding (*p* = 0.009, 0.035, 0.032).

Several studies have looked at the potential of NIRS in identifying gut injury with varying levels of success as we have summarised in Table 2 (15, 29–31, 34–39). There are a few studies to particularly highlight; a recent prospective cohort observational study (31) in appropriately grown preterm infants born at less than 30 weeks of gestational age demonstrated that cerebral oxygenation was significantly lower and FTOE significantly higher in infants who developed NEC across the first 10 weeks of life even when adjusted for potential confounding factors. This may suggest an underlying mechanistic relation between NEC and their worse neurodevelopmental outcome. The same study also found that splanchnic oxygenation was significantly lower and FTOE significantly higher in those infants who developed NEC compared to those without NEC (41, 42). NIRS continuous monitoring could therefore potentially help to identify infants with low cerebral or splanchnic oxygenation allowing earlier treatment and targeted neurodevelopmental interventions.

Studies using NIRS have shown that neonates with an acute abdomen, suggesting mesenteric ischaemia, have a lower Cerebral Splanchnic Oxygenation Ratio (CSOR), suggesting the CSOR could be used to identify gut injury (29). Fortune et al. (29) specifically looked at 40 neonates; 10 with acute abdomens (4 of these had NEC), 29 with normal abdomens and 1 with cerebral HIE. They found that the acute abdomen group had a significantly lower median CSOR value of 0.66 (0.45–0.69), *p* < 0.001 and that the CSOR had a 90% (56%–100%) sensitivity to detect splanchnic ischaemia in neonates; specifically, they reported that a CSOR of <0.75 was highly predictive of intestinal ischaemia and the need for surgical intervention. If continuous cerebral and splanchnic NIRS were being measured in an individual neonate, then should the CSOR drop this could alert clinicians to an increased severity of NEC and likely need for surgical intervention. However, a complicating factor in preterm infants is that the CSOR may be unreliable in the presence of intraventricular haemorrhage (IVH), which is a common complication of preterm birth. Infants with IVH would be expected to have impaired cerebral autoregulation which could affect the cerebral regional oxygenation (crSO_2_) and consequently the CSOR (15).

Schat et al. (36) examined 33 infants and compared the mean 8 h cerebral, liver and infraumbilical rSO_2_ and FTOE divided into infants with no NEC (*n* = 13), definite NEC (*n* = 20) and infants with uncomplicated (*n* = 10) and complicated NEC (*n* = 10) in the first 48 h after onset of symptoms that were felt to be suspicious for NEC. They found no significant differences in the first 24 h after onset of symptoms in rSO_2_ and FTOE between infants with no NEC and definite NEC. In preterm infants with complicated NEC, they demonstrated significantly lower cerebral, liver, and infraumbilical rSO_2_ and higher FTOE within 24 h after onset of symptoms compared with infants with uncomplicated NEC. Their results showed that crSO_2_ ≤ 71% and liver rSO_2_ ≤ 59% in the first 8 h after onset of symptoms predicted the onset of complicated NEC with a sensitivity of 1.0 and specificity of 0.8, and a sensitivity of 1.0 and specificity of 1.0, respectively. This suggests that NIRS could differentiate complicated NEC from uncomplicated NEC, but it did not differentiate between definite NEC and no NEC in preterm infants with clinical signs suspicious of NEC.

In addition to absolute changes in regional oxygenation more subtle changes have been identified in infants with NEC; in preterm neonates, a pattern of low srSO_2_ as well as high fluctuations in mesenteric oxygenation patterns were more pronounced in infants with NEC, especially before NEC onset (15, 27, 34). Cortez et al. (15) looked at 21 preterm infants born at less than 30 weeks gestation (2 excluded due to artifacts); who had abdominal NIRS placed from within 48 h of birth for the first 14 days of life. They demonstrated that daily mean srSO_2_ values decreased over the first 9 days of life (*p* < 0.0001), followed by increase from day 10–14 (*p* = 0.0061). The srSO_2_ was lower and FTOE higher in infants with feeding intolerance compared to those without (*p* = 0.0043). A higher srSO_2_ and variability was associated with a healthy gut (*n* = 17) and low srSO_2_ and decreased variability was noted in neonates with NEC (*n* = 2). NIRS has also been used to examine the relationship between red blood cell transfusion (RBCT) and gut injury to try and resolve the debate as to whether RBCT cause gut injury and NEC (27, 34) (Table 2). A recent case control study (*n* = 72) by Kalteren et al. (32) reported that RBCT were associated with concomitant signs of oxidative stress and intestinal injury as measured by raised levels of urinary 8-isoprostane and I-FABP respectively, along with lower variability in splanchnic oxygenation. The authors therefore postulated that this may represent the early pathogenetic process of transfusion-associated NEC. However, larger prospective trials are needed to substantiate conclusions as to whether this accurately reflects the onset of gut injury, as nearly all the current evidence is from non-randomised trials with only small study numbers.

### Practical issues with using NIRS as a potential biomarker for NEC onset

While there is some promising evidence in the literature, it is not conclusive and there are several issues that we must address first before we can consider NIRS as a potential biomarker for NEC onset and these include:

#### Lack of normal ranges

Most importantly, there is currently no large population-based normative data for regional saturation of various organs in infants which therefore subsequently limits its use in identifying gut injury, until we have established normal splanchnic oxygenation for infants of various gestation and postnatal ages (2). There have been numerous studies looking into establishing normal ranges using the two most common NIRS monitors and there is an impact of gestational and postnatal age, but these studies were all relatively small (9, 15, 43–47) (Table 1). Furthermore, most studies, do not include the micropremies between 22 and 24 weeks gestation, and it is this subgroup that are the highest risk of the associated morbidity and mortality with NEC and potentially will benefit the most from new technologies such as NIRS in monitoring their haemodynamic status. However, without first establishing normal ranges for such infants, detecting deviations from the norm and onset of possible NEC is problematic. Therefore, research should focus on the use of routine NIRS in extreme preterm infants and micropremies, so we can start constructing normative ranges. A recent study by van der Heid et al. (16) has brought us one step further as they conducted a relatively large NIRS study on preterm infants (*n* = 220, <32 w and/or <1,500 g BW) and showed that gestational age, postnatal age, and small-for-gestational age status affect regional splanchnic oxygen saturation and need to be taken into account when interpreting regional splanchnic oxygen saturations using NIRS. In their study they provided a model so that reference values for infant regional splanchnic oxygen saturation can be computed with their formula based on those factors.

A further issue that hinders the availability of normal ranges is that there are differing locations used for “abdominal” NIRS measurements; all of these locations have been used in the available studies and there is no consistent placement; some use liver, some infra-umbilical and some supra-umbilical. At present no one has conclusively confirmed which is the more reliable for detecting changes in abdominal regional oxygenation, although the infra-umbilical region is the most used (Table 1). Perhaps more importantly when trying to establish normal ranges the measurement location used significantly influences the regional saturation measurement obtained and there is no correlation between the three locations (48). Therefore, in addition to having to review the data from studies in relation to the device used, clinicians must also be aware of the measurement location that was used for the measurements.

#### Cost and training implications

To consider NIRS as a possible NEC biomarker we must remember that for this to work NIRS monitors would need to be used routinely, which would require a significant expenditure for each neonatal unit as well as the need for training medical and nursing staff on how to use it and interpret the readings. However, it is not inconceivable that with the ever-increasing body of evidence regarding the usefulness and clinical application of NIRS in predicting changes in gut blood flow and gut injury that it may be cost effective to use NIRS. By allowing earlier detection of NEC and initiation of treatment the impact of this disease could be reduced by decreasing the inevitable neonatal intensive care cot days and, more importantly, the long-term health burden for affected infants.

#### Safety of NIRS

NIRS is transcutaneous, non-invasive and does not cause harm to patients, therefore it is appropriate to continue to explore its usefulness in predicting NEC. The light intensities used are not harmful to the tissue, particularly when only used for short periods; furthermore NIRS is not known to cause skin burns even if applied for a longer period (48, 49). Current sensors for neonates are well tolerated due to their smaller size and because they are lined with a skin friendly adhesive. To provide further skin protection in extremely premature patients probes can be attached to a light-permeable skin barrier without interference with measurements (9).

#### Accuracy of NIRS measurements

Before its use in human studies, NIRS was initially used in laboratory and subsequently animal studies (50, 51). NIRS has been validated by using a newborn piglet model where the carotid, renal and mesenteric arteries were occluded and then re-perfused which caused fast, simultaneous changes in rSO_2_ of the affected end-organs (51). NIRS has since been validated in adult intensive care patients, those undergoing ECMO or cardiac surgery by comparing central venous samples with NIRS values (52–56). Its effectiveness and validity in measuring splanchnic oxygenation in preterm infants and the clinical implications of this were extensively examined in a recent systematic review article by Seager et al. (57). In a study by Menke et al. (58) two observers repeated a total of 500 NIRS measurements in 25 neonates and they performed a baseline measurement to assess the physiological variation in every neonate. They reported that inter-patient variance contributed most to the total variance, while the interobserver variance had the smallest effect. They demonstrated that crSO_2_ showed a good reproducibility, with an inter-measurement variance slightly but not significantly higher than the physiological baseline variation. Banerjee et al. measured gut oxygenation using NIRS in 71 preterm infants (59 receiving blood transfusion and 12 controls) and reported that NIRS is a reliable method of measuring changes in splanchnic tissue oxygenation and therefore gut tissue perfusion (59).

However, there are many factors that can affect the accuracy of the rSO_2_ obtained including the specific device used but also, unsurprisingly, given it is transcutaneous, the placement of the sensor is important. Placement of the sensor over fatty deposits, hair, bony protuberances, nevi, hematomas or broken skin, or application of pressure to the sensor may result in inaccurate readings. For measuring splanchnic oxygenation there are other factors that inherently affect the measurement reliability (48) because the gut is moving, can be filled with varying degrees of gas or stool and is near the stomach and bladder, all of which can affect results, and all of these are exacerbated in preterm infants given their size. It has previously been described that meconium interferes with the NIRS measurements (60) and depending on the gestational and chronological age of preterm infants, and whether an ileus is present or not, the amount of meconium present in the bowel will differ significantly. The currently available NIRS devices do not account for the absorption by stool and given the absorption spectra of meconium this can alter the optical signal from the NIRS device meaning that the measured regional oxygenation value will differ depending on the amount of meconium present in the bowel.

In addition, movements of the infant and consequently pull on the NIRS probes are more likely. The measurement accuracy of splanchnic oxygenation is affected by the amount of gaseous distension and faecal matter present in the abdomen which in a preterm is continually changing as they mature, due to the initiation of enteral feeds and depends on the level of respiratory support they are requiring; with non-invasive methods such as CPAP and Vapotherm being well known to increase abdominal distension.

NIRS is also susceptible to motion artefact caused by relative motion between the NIRS optical fibers and the skin of the region where they are placed. Relative motion will cause a rapid shift in the optical coupling between the NIRS optical fibers and the body area where the probe is placed (e.g., scalp or abdomen), which typically results in a period of high-frequency noise in the recorded NIRS data. In preterm infants this motion can be unavoidable; some critically ill preterm infants require high frequency oscillation ventilation (HFOV) which because of the chest “wobble” from the vibrations can be transmitted to the abdomen as well. Furthermore, even in more stable preterm infants movement can affect NIRS readings; NIRS motion artefact has been reported to be approximately 15% (61). Motion artifacts in NIRS data are often relatively easy to identify using a combination of observation of the subject during NIRS recording and visual inspection of the resulting data. However, there is no universal approach to whether remove or correct these (62). In most NIRS studies around 10% of infants are excluded because of motion artefacts (63, 64), but there have been recent studies demonstrating that you can avoid needing to exclude any infants due to motion artefact by using consistent probe placement (31, 47).

The accuracy of NIRS information is also affected by light scattering, the concentration of haemoglobin (Hb) and the level of melanin and bilirubin in the skin (65, 66). In neonatal intensive care, an obvious difficulty is in the first few days of life most preterm infants become jaundiced and many need phototherapy. Overhead phototherapy and the bilirubin level itself will affect the NIRS readings and, in some cases, the sensors will not work at all if the light is too bright near the sensors. Changes in Hb levels in preterm infants over the first few weeks of life have been shown to change NIRS measurements by a significant amount; 30%–50% (9, 65–67). Therefore, these factors would need to be considered when interpreting any NIRS measurements.

Finally it is well known that there is a high variability of the NIRS measurements in the abdominal region, although this has been shown to decrease over time (9, 47, 48, 68) This high variability limits the accuracy of NIRS measurements for both the detection of NEC onset and establishing normal ranges. This higher variability seen at the abdominal region is to be expected given the inherent limitations we have already discussed, as there is a plethora of factors which can alter its accuracy and cause high variability, including gut peristalsis, faecal and air content, as well as the impact of nursing cares including nappy changes. There are emerging techniques looking at ways to improve the reliability and accuracy of NIRS measurements by reducing this variability and Isler et al. (60) recently published their study where they examined the optical properties of faeces of preterm infants to enable a more accurate measurement, considering the effect of stool on the NIRS measurements. Therefore, it is conceivable that in the future corrections such as this will be commercially available for all NIRS devices and subsequently allow for more accurate NIRS abdominal regional oxygenation measurements.

### Summary of effectiveness

NIRS is non-invasive and safe (48, 49, 69); but the accuracy of splanchnic oxygenation measured is affected by the structure and content of the bowels (48). NIRS can detect alterations in splanchnic oxygenation and perfusion, potential allowing earlier recognition of bowel ischaemia and gut injury such as NEC than currently used methods. However, its widespread application is limited given that current literature evidence has involved mostly very small studies, using both term and preterm infants of various gestations and postnatal ages as well and with varying NIRS monitors (2). One limitation of NIRS is that repeated measures within subject standard deviation is about 5% to 6% (70) and there is a methodological bias between sensors from the two most commonly used monitors- INVOS-5100 and NIRO-300 (71) and therefore attention needs to be paid to the type of sensor used and the device used when interpreting any regional oxygenation results. Because authors report studies on regional oxygenation using different NIRS monitors the emphasis should be on using NIRS to report trends in regional oxygenation (72).

## Conclusion

Despite advances in neonatal care, the severity of NEC and its morbidity and mortality remain high. As a neonatal community our research must focus on identifying this devastating disease prior to symptom onset.

Continuous splanchnic NIRS monitoring may benefit the clinical outcomes of preterm infants because NIRS has the potential to alert clinicians to when relative regional hypoxia occurs for an individual neonate allowing time for prompt interventions to ameliorate their hypoxia, and potentially reduce their long-term impairments. However further trials involving larger numbers of infants are needed to examine if NIRS monitoring coupled with intervention can improve outcomes. Perhaps the real answer is using a machine learning algorithm combining routine clinical, laboratory and gut tissue biomarkers along with NIRS on each individual preterm neonate in intensive care; therefore, we need larger scale observational studies and randomised trials combining NIRS with the more successful tissue biomarkers including intestinal fatty acid binding proteins (IFABPs), which are the most studied gut biomarker, and other clinical parameters to review their effectiveness in predicting NEC onset in larger numbers of infants.

While there is currently no conclusive evidence regarding NIRS and its ability to predict NEC there are promising small studies and the option of having a non-invasive method of monitoring gut injury is an exciting concept and justifies further research in this area.
